# A Korean multi-center, real-world, retrospective study of first-line pazopanib in unselected patients with metastatic renal clear-cell carcinoma

**DOI:** 10.1186/s12894-016-0163-5

**Published:** 2016-08-02

**Authors:** Moon Jin Kim, Se Hoon Park, Jae-Lyun Lee, Se-Hoon Lee, Su Jin Lee, Ho Yeong Lim

**Affiliations:** 1 Division of Hematology–Oncology, Department of Medicine, Samsung Medical Center, Sungkyunkwan University School of Medicine, Seoul, Korea; 2 Department of Oncology, Asan Medical Center, University of Ulsan College of Medicine, Seoul, Korea; 3 Department of Internal Medicine, Seoul National University Hospital, Seoul, Korea; 4 Department of Medicine, Myongji Hospital, Goyang-si, Gyeonggi-do Korea

**Keywords:** Clear-cell carcinoma, Renal cell carcinoma, Pazopanib, First-line

## Abstract

**Background:**

The efficacy and/or tolerability of pazopanib in patients with metastatic renal cell carcinoma (mRCC) have been found to differ in Western and Asian populations. This retrospective multicenter study analyzed the results of first-line pazopanib treatment in 93 consecutive patients with mRCC who were treated at the medical oncology departments of three tertiary cancer centers in Seoul, Korea.

**Methods:**

The decision to administer pazopanib as first-line therapy was at the discretion of the treating physician in all patients with mRCC. Patients enrolled in clinical trials were excluded to ensure that the results would reflect real-world outcomes representative of daily clinical settings. All patients received 800 mg/day pazopanib. Outcomes included response rate, progression-free survival (PFS), overall survival (OS), and safety.

**Results:**

The 93 patients included72 (77 %) male and 21 (23 %) female individuals, of median age 65 years (range, 19–84 years). The median number of metastatic sites per patient was two (range, 1–5), with the lungs being the most frequently involved site. Most patients had favorable (*n* = 46) or intermediate (*n* = 36) risk as determined by Memorial Sloan Kettering Cancer Center criteria. Pazopanib was generally welltolerated: the major hematologic adverse effect was grade 1/2 anemia (14 %); and the most frequently observed non-hematologic toxicity was grade 1/2 mucositis (22 %), followed by hair discoloration and hypertension. Of the 93 patients, three (3 %) showed complete response, 52 (56 %) showed partial response, and 21 (23 %) showed stable disease, making the objective response rate 59 % and the disease control rate 82 %. At a median follow-up of 21 months, the estimated median PFS and OS were 12.2 months (95 % confidence interval, 7.1–17.4 months) and 21.9 months (95 % confidence interval, 12.9–30.9 months), respectively.

**Conclusions:**

In this retrospective study, first-line therapy with pazopanib demonstrated clinically relevant efficacy and tolerability in unselected real-world Korean patients with mRCC. OS and PFS of these Korean patients were similar to those reported in phase III trials.

## Background

Renal cell carcinoma (RCC) is the most common type of kidney cancer [1], with clear cell carcinoma being the most common subtype. Because approximately 30 % of patients present with primary metastatic disease and one-third of patients have recurrent metastatic disease after nephrectomy with curative intent [2], over 50 % of all patients with RCC require systemic therapy during the course of their disease. RCC has been found refractory to conventional systemic chemotherapeutic agents and radiotherapy. Clear cell RCC represents a unique clinical setting for the application of antiangiogenic therapy, in that targeting of angiogenesis through pathways involving vascular endothelial growth factor receptor (VEGFR) and mammalian target of rapamycin has produced robust clinical effects and revolutionized the treatment of metastatic RCC [3]. Multi-targeted tyrosine kinase inhibitors (TKIs) against VEGFR, including sunitinib [4], sorafenib [5], and pazopanib [6], have improved progression-free survival (PFS) and/or overall survival (OS) when compared with interferon and/or supportive care in patients with metastatic RCC.

Current guidelines recommend the use of sunitinib, pazopanib, or bevacizumab plus interferon as first-line treatment for favorable- or intermediate-risk patients with metastatic, clear cell RCC [7]. Because of their oral route of administration and more favorable toxicity profiles, sunitinib and pazopanib are the most widely administered TKIs in this setting. The choice of a first-line regimen is important, because not all patients are eligible for salvage therapy, providing an obvious rationale for administering the most effective initial treatment. For example, in the large phase IIICOMPARZ (COMParing the efficacy, safety and tole Rability of paZopanib versus sunitinib) trial [8], 1,110 patients with metastatic RCC were randomly assigned to receive either sunitinib or pazopanib. Baseline patient and disease characteristics were well balanced between the two arms. Based on independent review by blinded radiologists, median PFS was 8.4 months in the pazopanib group versus 9.5 months in the sunitinib group, with the hazard ratio (HR) of 1.05 (95 % confidence interval [CI] 0.90**–**1.22) being within the acceptable boundaries of non-inferiority. Although pazopanib and sunitinib showed similar efficacy, safety and quality-of-life (QOL) profiles favored pazopanib. These findings were confirmed in the subsequent randomized, double-blind, crossover PISCES (PazopanIb versus Sunitinib patient preference Study) study, which compared QOL and patient preference in patients treated with pazopanib or sunitinib [9].

Based on these results, pazopanib has become one of the most frequently used VEGFR TKIs for metastatic RCC in Korea. However, the choice of a first-line TKI for an individual patient can be a clinical dilemma. Factors that must be considered include the experiences of the treating oncologists, the activities of the TKIs, and the potential toxicities of these agents, especially for patients with symptoms or decreased performance status. Moreover, the efficacy and/or tolerability of pazopanibhas been found to differ in Western and Asian populations. For example, subset analysis of the COMPARZ trial [10] found that, in Asian patients, median PFS was longer with sunitinib than with pazopanib (11.1 versus 8.4 months, HR 1.07, 95 % CI 0.81**–**1.42), although the difference was not statistically significant. In addition, Asian and non-Asian RCC patients experienced different adverse events: hematologic toxicities, hypertension, and hand-foot-syndrome were more frequently observed in Asian patients, whereas fatigue and gastrointestinal symptoms were more frequent in non-Asians, regardless of treatment arm. Patients in phase III clinical studies, including COMPARZ, were selected on the basis of a fairly preserved performance status and normal organ function. However, RCC is a highly aggressive disease, which often shows rapid progression and clinical decline; therefore, the clinical trial population may not be representative of all patients seen in real-world daily oncology practice. Based on these considerations, this multi-center retrospective studywas designed to evaluate the efficacy and safety of first-line pazopanib in Korean patients with RCC.

## Methods

The medical records of 93 consecutive adults with histologically proven mRCC and predominant clear cell histology who were treated with pazopanib as first-line TKI therapy in 2012 were retrospectively reviewed. The decision to treat with pazopanib was solely at the discretion of the treating oncologist. Patients enrolled in clinical trials were excluded to ensure that the study population reflected daily clinical practice in our institutions. Patients were also excluded if they had: (1) received prior chemotherapy or anti-angiogenic therapy for advanced or metastatic disease, (2) 100% non-clear cell carcinoma,(3) another malignancy within 5 years, and (4) inappropriate laboratory findings or severe comorbid illness during treatment with the standard dose of pazopanib (800 mg/day). This study was approved by the Institutional Review Boards of Samsung Medical Center, Asan Medical Center, and Seoul National University Hospital. Written informed consent was provided by all patients prior to starting pazopanib treatment, according to institutional standards.

All patients were treated with 800 mg/day pazopanib, administered orally without interruption. Supportive care, including the administration of blood products and analgesics, was provided if judged appropriate by the treating physician. Before treatment with pazopanib, patients had a complete history taken and underwent complete blood counts and serum chemistries, chest x-rays, and computed tomography scans of all involved sites. Patients were assessed every 4 weeks because pazopanib therapy was repeated at this interval. Therapy was continued until objective disease progression per Response Criteria in Solid Tumors [11], unacceptable toxicity or deterioration of hepatic function, or patient refusal. Baseline characteristics and outcome data were collected using a uniform case report form. Clinical and laboratory parameters collected at the time of starting pazopanib treatment included, but were not restricted to, those described inthe Memorial Sloan Kettering Cancer Center (MSKCC) [2] and Heng [12] prognostic criteria: age, sex, Eastern Cooperative Oncology Group (ECOG) performance status, the presence of other histologic types than clear cell carcinoma, previous nephrectomy, previous cytokine therapy, neutrophil count, platelet count, hemoglobin, serum lactate dehydrogenase, corrected serum calcium, time between diagnosis and TKI therapy, and sites of metastases. Responses were evaluated every 8 weeks by chest and abdominopelvic computed tomography or by the same tests that were used to stage initial tumors. Adverse events were graded according to the National Cancer Institute criteria (CTCAE v4). Causes of death and discontinuation of therapy were evaluated by a structured review of medical records.

The primary endpoint was PFS, defined as the time between pazopanib initiation and the date of documented disease progression or death, whichever occurred first. Secondary endpoints included OS, response rate, and toxicity profile. OS was defined as the time from the first day of pazopanib administration to death from any cause. PFS and OS were calculated using the Kaplan–Meier method. The impact of baseline parameters on PFS and OS was assessed using a Cox proportional hazards model. Laboratory parameters and age were recorded as continuous variables and were evaluated as both continuous and categorical variables. The potential presence of interaction effects between baseline parameters was tested by defining product terms for the respective factors in a regression model. All P values were two-sided, with *P* < 0.05 indicating statistical significance. All statistical analyses were performed using the SPSS package (version 16.0) and R for Windows v2.11.1 software (R Core Team, Vienna, Austria; http://www.Rproject.org).

## Results

Medical records were collected from 93 consecutive patients who were treated with first-line pazopanib at three tertiary Korean cancer centers between January and December 2012. Patient characteristics are given in Table 1. Of the 93 patients, 72 (77 %) were male individuals and 21 (23 %) were female individuals. Nine (10 %) patients had previously been treated with cytokine therapy, the majority with high-dose interleukin-2. Eighty-two (88 %) patients had favorable or intermediate MSKCC risk scores, whereas 11 (12 %) had an ECOG performance status of 2 or higher. Approximately 60 % of the patients had two or more metastatic disease sites, mostly involving the lungs and lymph nodes. At the time of data collection, corresponding to a median follow-up of 21 months, 82 patients (88 %) had discontinued pazopanib treatment and 40 (43 %) had died.

**Table 1:** Baseline characteristics

	No. of patients	Percent
Age, years		
Median (range)	65 (19–84)	
Sex		
Male	72	77
Female	21	23
ECOG performance status		
0	4	4
1	78	84
2 or higher	11	12
Comorbid illness		
Any	52	
Diabetes	23	
Hypertension	42	
Obstructive lung disease	11	
Renal insufficiency	3	
Histology		
Clear cell carcinoma (100 %)	87	93
Mixed	6	7
Time from diagnosis to treatment, months		
Median (range)	14 (0–83)	
Prior therapy		
Nephrectomy	74	80
Cytokine immunotherapy	9	10
Radiotherapy	6	7
Laboratory findings (mean, SD)		
Hemoglobin, g/dL	12.6 (2.0)	
Corrected calcium, mg/dL	9.3 (0.7)	
Lactate dehydrogenase, U/L	372 (243)	
Number of metastatic site(s)		
Median (range)	2 (1–5)	
Sites of metastases		
Lung	66	
Lymph nodes	21	
Bone	18	
Liver	11	
Brain	17	
MSKCC risk		
Favorable	46	49
Intermediate	36	39
Poor	11	12
Heng score		
Favorable	40	43
Intermediate	38	40
Poor	15	17

The 93 patients received a total of 1,086 4-week cycles of pazopanib (median 12, range 1–34). In 32 cycles (0.03 %), involving 12 (13 %) of the 93 patients, doses were reduced by 25 %. The reasons for dose reduction were available for 11 patients, the most common being non-hematologic toxicities, including fatigue (4/11, 36 %), gastrointestinal discomfort (3/11, 27 %), and hypertension (2/11, 18 %). In addition, two (18 %) of these 11 patients required dose reductions for hematologic toxicities, both being grade 3 thrombocytopenia. The most common reason for therapy discontinuation was disease progression, Overall, first-line pazopanib was generally well tolerated, with hypertension, anemia, and oral mucositis being the most commonly observed toxicities (Table 2). Twelve (13 %) patients experienced transient and reversible elevation of liver function tests (LFTs); of these, one patient had early liver cirrhosis owing to hepatitis B virus infection and three were heavy consumers of alcohol, whereas the other eight had no underlying hepatic diseases. All 12 patients received ursodeoxycholic acid to normalize LFTs, but none requirement pazopanib dose modification or delay. Abnormal LFTs were all normalized within 3 months. Two patients died of causes for which we could not completely rule out a relationship to pazopanib. One patient died of a pulmonary thromboembolism during the middle of the second cycle of pazopanib treatment, with no clinical evidence of progression. The second patient, who had multiple lung and lymph node metastases, died of interstitial pneumonitis after 12 months of clinical response to pazopanib. Although the size of involved lymph nodes remained unchanged, the possibility of disease progression could not be completely excluded.

**Table 2:** Maximum grade toxicity recorded per patient (*n* = 93)

	Grade 1–2		Grade 3–4	
	N	%	N	%
Anemia	19	20	1	1
Neutropenia	2	2	0	
Thrombocytopenia	12	13	0	
Nausea	3	3	0	
Vomiting	2	2	0	
Anorexia	6	7	0	
Stomatitis	20	22	2	2
Diarrhea	15	16	3	3
Fatigue	9	10	1	1
Skin	11	12	0	
Hepatic	12	13	0	
Hypertension	22	24	0	

Of the 93 patients, two could not be evaluated for response to pazopanib because of the absence of measurable lesions or early discontinuation of therapy. Three (3 %) patients showed a complete response (CR) and 52 (56 %) had a partial response, making the objective response rate 59 % (95 % CI 45–65 %). In addition, 21 (23 %) patients had stable disease, making the disease control rate 82 %. Patients with poor MSKCC risk scores were significantly less likely to respond to pazopanibthan patients with favorable or intermediate risk scores (*P* = 0.03). Response rate was not significantly influenced by age, sex, number and site of metastases, or baseline laboratory parameters.

**Fig. 1:**
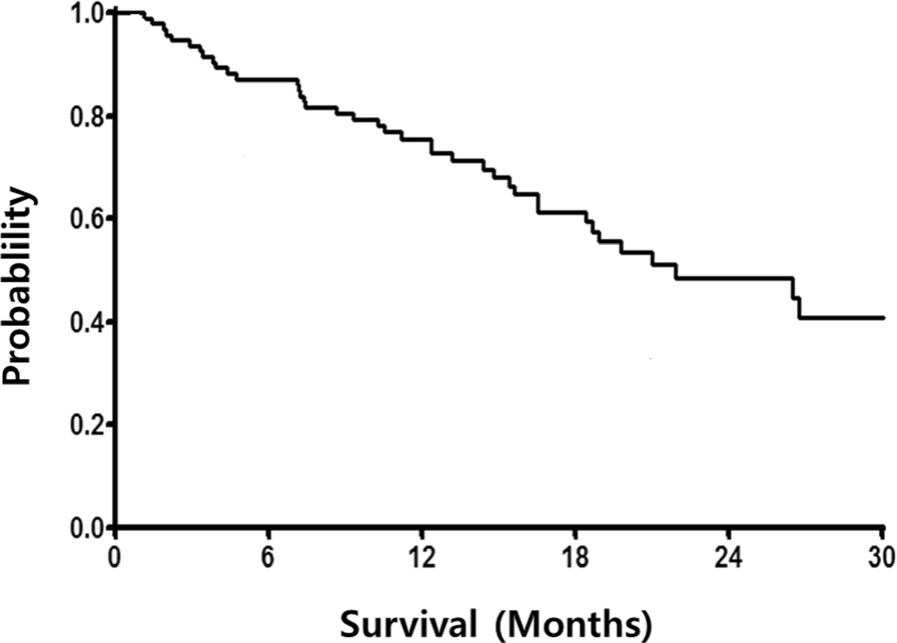
Kaplan–Meier analysis of overall survival of all patients

**Fig. 2:**
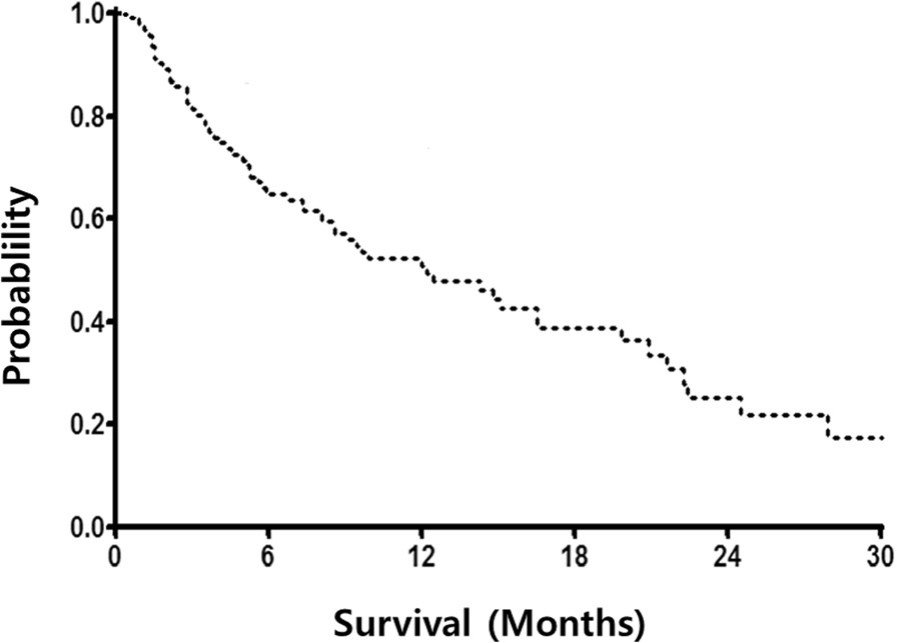
Kaplan–Meier analysis of progression-free survival of all patients

**Fig. 3:**
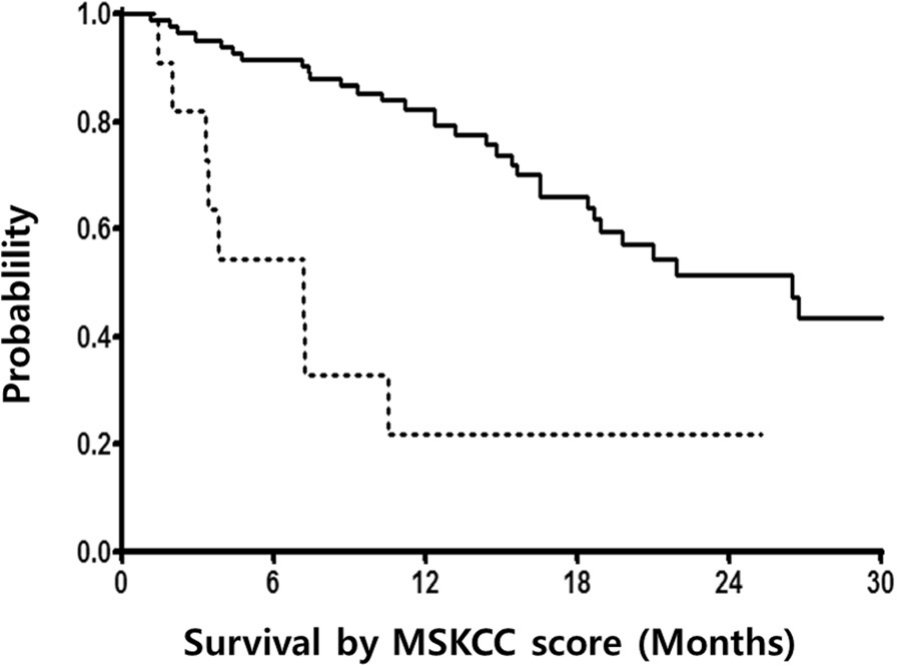
Kaplan–Meier analysis of overall survival according to MSKCC risk scores. Overall survival was significantly greater in patients with favorable or intermediate risk scores (solid line) or a poor risk score (dotted line) (hazard ratio, 4.07; 95 % confidence interval, 1.84–9.01; *P* = 0.001)

The median OS forthe 93 patients analyzed in the study was21.9 months (95 % CI 12.9–30.9 months, Fig. 1) and the median PFS was 12.2 months (95 % CI 7.1–17.4 months, Fig. 2). Median PFS was significantly shorter for patients with poor risk than for patients with favorable or intermediate MSKCC riskscores (2.4 months [95 % CI 2.0–2.8 months] vs. 12.5 months [95 % CI 8.3–16.7 months], *P* < 0.0001). Similarly, median OS was shorter for patientswith poor risk than for patient with favorable or intermediate MSKCC risk scores (7.2 months [95 % CI 1.5–12.9 months] vs. 26.5 months [95 % CI 18.9–34.1 months], *P* < 0.001; Fig. 3). This model suggested that mRCC patients with poor MSKCC risk scores had a 4-fold higher risk of death than patients with favorable or intermediate risk scores. We also tested whether OS was altered by interactions with poor MSKCC risk scores and other clinical characteristics by entering the first-level interaction term between these variables into separate multivariate models; however, the results were not significant.

For exploratory purposes, we compared OS according to the clinical response to pazopanib. OS was longer in responders (26.7 months) than in non-responders (18.6 months), although this difference was statistically insignificant (*P* = 0.106).In contrast, OS was significantly longer in patients who did than did not achieve clinical response or stable disease (26.5 months vs. 14.8 months, *P* = 0.007). After the failure of pazopanib treatment, 37 % of the patients received second-line therapy, mostly with everolimus (*n* = 30) or another VEGFR TKI (*n* = 4).

## Discussion

This retrospective study on a limited number of Korean RCC patients showed that first-line therapy with pazopanib was both well tolerated and effective, regardless of performance status or the number of metastases. Pazopanib achieved an objective response and stable disease in 59 and 23 % of patients, respectively. The estimated median PFS and OS were 12.2 months (95 % CI, 7.1–17.4 months) and 21.9 months (95 % CI, 12.9–30.9 months), respectively. These results compared favorably with the outcomes of large phase III trials [6, 8]. Although this study was retrospective in nature, its results indicate that Korean patients with metastatic RCC may derive clinically relevant benefits from pazopanib. Most adverse events were transient and self-limiting, and there were few severe non-hematologic toxicities, with grade 3 or 4 stomatitis or diarrhea occurring in only 2–3 % of patients.

VEGFR TKIs have been the mainstay of treatment of patients with clear cell mRCC. Because the main goal of treatment remains palliation, the choice of TKI is based not only on efficacy, but on the consideration of other parameters, include patient preference, relief of symptoms, and/or QOL. The phase III COMPARZ trial, a direct head-to-head comparison between sunitinib and pazopanib indicated that both TKIs are effective and feasible for the first-line treatment of patients with mRCC [8]. Although these two TKIs had similar efficacy, safety and QOL data favored pazopanib. Interestingly, subgroup analysis of patients in the COMPARZ trial showed marked geographic and/or ethnic differences in toxicity profiles and efficacy. In Asian patients, the median PFS was longer with sunitinib (11.1 months) than with pazopanib (8.4 months) [10], although the difference was not statistically significant (HR 1.07, 95 % CI 0.81–1.42). One possible explanation is that the incidence of adverse events differs according to ethnicity. The discontinuation rate owing to adverse events in the COMPARZ trial was 20 % for sunitinib and 24 % for pazopanib. Asian RCC patients experienced hematologic toxicities, hypertension, hand-foot syndrome, liver dysfunction, and proteinuria more frequently than non-Asian patients, whereas fatigue and gastrointestinal symptoms were observed less frequently in Asian patients, regardless of the treatment arm.

In contrast to the COMPARZ subgroup results [10], sunitinib is thought to be less well tolerated by Asian than Western RCC patients, leading to the widespread administration of reduced suboptimal doses of sunitinib to Asian patients [13]. Because of the clear-cut relationship between drug exposure and efficacy [14], maintaining adequate TKI doses is essential to optimize treatment outcomes in Asian RCC patients [15]. These ethnicity-based differences may be owing to chance, to differences in tumor biology between Asian and Western patients, or to a pharmacogenomic difference in drug metabolism leading to differences in drug exposure.

It is difficult to determine whether pazopanib or sunitinib is more effective in Asian patients with mRCC, even when including results from the analysis of Asian subpopulations in a sunitinib expanded access program [16]. Choice of a first-line TKI regimen for individual patients with mRCC requires careful considerations of eachpatient’s disease status, symptoms, general condition, and preference. The results of the present study indicate that pazopanib is a reasonable option for Korean mRCC patients and that poor MSKCC risk score is a significant predictor of reduced survival. Studies are underway to identify possible molecular markers, as well as specific genotypic variations in different ethnicities, which may be linked to responsiveness or resistance to VEGFR TKIs.

The strength of the current study includes its multicenter nature and the enrollment of patients who were treated with pazopanib as routine clinical practice to avoid selection bias. The patients included in this study were those treatedat academic tertiary cancer centers, reflecting real-world experience with first-line pazopanib. This population may differ from those in clinical trials and may be more relevant to those seen by the clinicians in daily practice. That is, the results of this study may better reflect real-world outcomes that may not necessarily be seen in randomized controlled trials of selected patients.

This study also had several limitations, including its retrospective nature, which may have introduced selection bias and issues regarding missing data. However, selection bias can be minimized by evaluating a consecutive series of patients, as in the current study. Other limitations include the lack of central radiology review, the use of various imaging modalities, and different intervals between scans; however, these variations better reflect the real-world clinical experience of oncologists who administer targeted therapy. Finally, the lack of a comparative arm precludes our ability to determine whether pazopanib is superior, or at least equivalent, to other agents such as sunitinib.

## Conclusions

The results obtained in the present study suggest that pazopanib is active and safe in the first-line treatment of Korean patients with mRCC. Better patient selection may improve clinical outcomes of mRCC patients in a first-line setting. Emerging clinical data and greater knowledge of the disease may further guide the development of individualized treatment regimens for patients with mRCC.

## Abbreviations

mRCC, metastatic renal clear-cell carcinoma; OS, overall survival; PFS, progression-free survival; VEGFR, vascular endothelial growth factor receptor
